# Chemical and Sensory Profile of Grape Distillates Aged in *Quercus alba* Casks Previously Used for Sherry Wine or Brandy

**DOI:** 10.3390/molecules29225303

**Published:** 2024-11-09

**Authors:** Daniel Butrón-Benítez, Manuel J. Valcárcel-Muñoz, M. Valme García-Moreno, M. Carmen Rodríguez-Dodero, Dominico A. Guillén-Sánchez

**Affiliations:** 1 Departamento de Química Analítica, Facultad de Ciencias, Instituto Investigación Vitivinícola y Agroalimentaria (IVAGRO), Universidad de Cádiz, Campus Universitario de Puerto Real, 11510 Puerto Real, Cádiz, Spain; daniel.butron@uca.es (D.B.-B.); maricarmen.dodero@uca.es (M.C.R.-D.); 2 Bodegas Fundador S.L.U., C/San Ildefonso, n° 3, 11403 Jerez de la Frontera, Cádiz, Spain; 3 Oeno R&D FL, 11402 Jerez de la Frontera, Cádiz, Spain; mjc.valcarcel@gmail.com

**Keywords:** ageing, sherry cask, brandy cask, grape distillates, marc distillate, congeners, sensory analysis, factorial analysis, LDA

## Abstract

This work investigates the influence of oak-cask ageing on the chemical composition and sensory profile of a variety of grape distillates. Wine spirit, wine distillate, neutral alcohol, and grape marc distillate were investigated. It is known that the characteristics of the ageing casks may have a considerable impact on the ageing process, so casks that had previously contained some type of sherry wine, sherry cask^®^, and casks that had previously contained brandy were studied. The results showed that ageing in either type of cask resulted in significant changes regarding volatile compound composition and a noticeable increase in phenolic and furfural compound content. Furthermore, sherry casks^®^ contributed with sherry wine characteristic compounds that enriched the aromatic profile of the distillates, such as a greater increase in ethyl esters of organic acids. A less noticeable evolution was exhibited by the distillates with higher levels of congeners (wine spirit and grape marc distillate) when compared to wine distillate or neutral alcohol, where changes due to ageing were more evident. The sensory analysis confirmed that ageing significantly modified the organoleptic characteristics of all the distillates, with an increasing perception of certain notes such as oak, vanilla, spicy, and vinous when aged in sherry cask^®^.

## 1. Introduction

Current European regulations define spirit beverages as alcoholic drinks intended for human consumption that exhibit specific organoleptic qualities and are obtained through the distillation of agricultural products [1]. They also state that ethyl alcohol of agricultural origin is obtained through the distillation of fermented agricultural products and has the characteristic aroma of the raw material used (such as grapes).

Wine spirit is defined as a spirit obtained through the distillation of wine, fortified wine, or wine distillate (previously distilled to less than 86% alcohol by volume (ABV)). No maturation period has been established, but one may apply as long as it complies with the minimum requirements established for brandies. Brandy is a spirit made from wine spirit (distillate at <86% ABV, with a high concentration of less volatile than ethanol congeneric compounds—mainly higher alcohols and esters), which may be blended with wine distillate (distillate at <94.8% ABV, rich in more volatile than ethanol congeneric compounds—mainly acetaldehyde, acetal, and ethyl acetate) [2,3]. This blend can be made as long as the alcohol coming from the distillate does not exceed 50% of the alcohol content in the final product. The volatile compound content must come exclusively from wine distillation and must be at least 125 g/hL 100% vol. alcohol for brandy, and 150 g/hL 100%vol. alcohol for Brandy de Jerez [1,4]. Brandy must be aged for at least 6 months in oak casks of less than 1000 L volume [1]. Brandy is produced all over the world, with the most famous ones produced in France (Cognac and Armagnac) [5]. In Spain, brandy is produced in various regions, with the main one being Brandy de Jerez, which is characterized by its traditional ageing system [4].

Grape marc distillate is a spirit obtained by distilling fermented marc, i.e., the solid parts of grapes (skins, pips, and stems) that are obtained through pressing [1]. They must be obtained at less than 86% ABV and the first distillation is to be carried out in the presence of the marc itself. These solid parts of the grapes give rise to distillates with a large content of compounds at relatively high concentrations, some of which are considered undesirable, such as methanol. Grape marc distillates are produced in most European wine-producing countries (Spain, Italy, France, and Portugal). In Spain, *Orujo* is the grape pomace distillate produced in Galicia, a traditional winery region in Northwest Spain [6]. Despite the positive impact that ageing in wooden barrels has on the spirits, it is not mandatory in the case of grape marc distillate and for this reason, only a few distilleries implement this ageing process for a small part of their production. According to a report by the Ministry of Agriculture in Spain, less than 1% of the total *Orujo* marketed in 2023 had been aged [7].

The aromatic profile of distillates is marked by their volatile composition. Volatile compounds derive from different sources [8]. Firstly, they are derived from the raw material, where the state and variety of the grape used has a strong influence on the final aroma. Secondly, from the fermentation process, metabolites are generated as a result of the activity of yeasts and bacteria. Thirdly, the compounds originate from the reactions that occur during the distillation process. Finally, in the case of distillates that are aged in wooden casks, the volatile compounds are extracted from the wood during the ageing stage [2,9,10].

The changes that the distillates experience, during the ageing process, are conditioned by numerous factors, including the characteristics of the wood casks used. These characteristics include the botanical origin of the wood, the volume of the barrel, the manufacturing method, and the previous usage of the cask. Although there are studies that have chemically and sensorially analyzed distillates aged in oak casks, and in some cases in casks made of other woods, studies on the ageing in casks used for other purposes, such as sherry Cask^®^, are very scarce [11,12]. Sherry Casks^®^ are oak casks that have previously contained sherry wine that confer the distillates with the characteristic profile of this wine during the ageing process. These casks are mandatory for the production of Brandy de Jerez but are also increasingly used by producers of high-quality whiskeys or rums. During the ageing process, sherry casks^®^ give off compounds from the wood as well as from the wine that has been previously contained in them over their seasoning stage. This contributes to the complexity and aromatic richness of the final distillates [8]. Oloroso sherry is one of the wines that can be used to season sherry casks^®^ and we have used it for this research. Oloroso sherry is an oxidative ageing dry wine with 18–20% ABV and a high content of higher alcohols, aldehydes, esters, organic acids, polyalcohols, phenolic, and furfural compounds that come from the grape, the fermentation, and the casks oak wood [13]. The unseasoned casks had previously contained brandy, characterized by its high content of higher alcohols, esters, and aldehydes (acetaldehyde) [14]. The aromatic profile of the final brandy was characterized by the complexity of its compounds, with aldehydes and higher alcohols, as well as alcohols and fatty acid esters that provided the fruity aromas of distillates. The ageing process enhances the aromatic profile of brandy, as phenolic compounds emerge to contribute with new notes such as toasted, vanilla, or spicy, while notes of bitterness and astringency become part of its flavor profile [15].

This research intends to make a valuable contribution to the spirits industry by comparing, at an industrial scale, the evolution of distillates during ageing, and how clearly different aromatic profiles can be obtained under similar conditions. Additionally, it intends to gain some insight into the influence that the cask features may have on the distillates. Therefore, the present work aims to assess the impact of ageing on the volatile composition and sensory profile of a variety of grape distillates. Specifically, four distillates have been studied (wine spirit, wine distillate, neutral alcohol of vinous origin, and grape marc), all of them were aged for two years using two types of casks: sherry casks^®^ (SC) and casks that had previously contained brandy (BC). Their aromatic profiles were determined through the chromatographic analysis of their volatile and phenolic compound content before and after 1 and 2 years of ageing. The samples were also subjected to a sensory evaluation.

## 2. Results and Discussion

In the present work, the results are expressed as the aggregate corresponding to each family of compounds studied; the tables showing the content of the individual analytes are included in the Supplementary Materials.

### 2.1. Volatile Compounds

#### 2.1.1. Aldehydes

**Figure 1:**
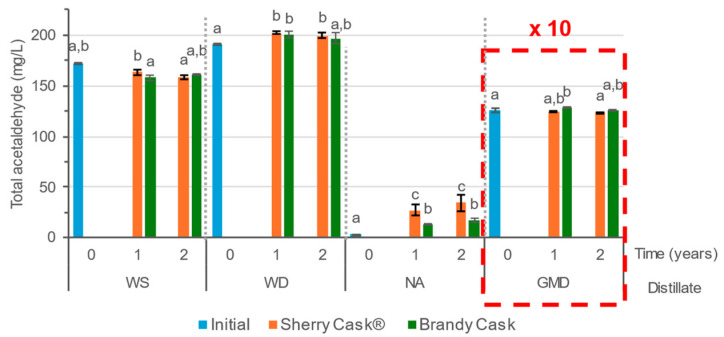
Total acetaldehyde in the initial wine spirit (WS), wine distillate (WD), neutral alcohol (NA), and grape marc distillate (GMD) and after ageing for 1 and 2 years. GMD values should be multiplied by a factor of 10. Different letters indicate that a significant difference has been obtained from the Tukey HSD test (*p* < 0.05).

The concentration of total acetaldehyde is defined as the sum of the acetaldehyde present in the distillates in its molecular form as well as that combined with ethanol to form its acetal, acetaldehyde-diethyl acetal (1,1-diethoxyethane), expressed as mg/L acetaldehyde (Figure 1). Both acetaldehyde and acetal are produced during wine fermentation—first, acetaldehyde is formed, and then, as the alcohol in the medium increases, acetylation occurs until equilibrium is reached. During the distillation, most of the acetaldehyde, which is found in the head fraction, is separated. A part remains in the core fraction of the distillate, where a higher concentration of acetal is formed because of the greater presence of ethanol in the medium. Depending on the equipment and the distillation process used, the separation of heads is almost total in the case of neutral alcohol, less for wine distillates and spirits, and even less for grape marc distillate. Therefore, the total acetaldehyde values of the initial distillates before ageing (time 0) depend on the conditions established for their fermentation and distillation [16,17]. Later on, during the ageing process, no clear trends were observed. In the case of wine spirit (WS), no significant differences between the samples aged in either brandy cask (BC) or sherry cask^®^ (SC) were registered after 2 years of ageing. On the other hand, the wine distillate (WD) and the neutral alcohol (NA) exhibited some increments with regard to acetaldehyde content during their ageing process in either BC or SC. It is worth noting that a greater increase took place in NA when aged in SC. This increase was determined by the oxidation of ethanol into acetaldehyde, and no differences were observed between the 1st and the 2nd year of ageing, which suggests that the processes involving acetaldehyde, such as evaporation and oxidation, were already in equilibrium [11,17]. The variations observed during the ageing process of GMD were not significant. This could be attributed to the fact that the evolution of both acetaldehyde and its acetal is conditioned by their high initial concentrations.

Benzaldehyde is an aromatic aldehyde that has an influence on the aroma of the distillate [8]. In wines, it comes from the degradation of the lignin from the grape seeds and its presence in the distillate will depend on the distilling conditions [10,16]. It has been observed that the initial distillates WS, WD, and NA do not initially contain benzaldehyde (Table 1), as there is no contact time with the solid parts of the grapes during the fermentation of the white wines used, and the small amount that is formed during the distillation is completely separated. On the other hand, because of the characteristic elaboration process of GMD, it contains high levels of benzaldehyde, since both the fermentation and the first distillation are carried out in the presence of the grape lees (pips and skins pressed) [1]. During the ageing process, benzaldehyde appears in the distillates as a result of the degradation of the lignin present in the wooden casks [10]. WS and WD do not present differences in their benzaldehyde concentration when aged in either BC or SC, and there are no significant variations between the first and second years of ageing. In the case of NA, benzaldehyde content increases over the first and the second year and reaches its highest concentrations after 2 years of ageing in BC. With regard to benzaldehyde content in GMD, a decrease is observed when aged in BC and a slight increase when aged in SC, exhibiting considerably higher values with respect to those of the rest of the distillates (>3.20 mg/L).

#### 2.1.2. Alcohols

The methanol content in alcoholic beverages is of utmost importance because of its toxicity (the maximum legal limit for agricultural neutral alcohol is 0.3 g/L 100% ABV, for distillate and wine spirits 2.0 g/L 100% ABV, and for marc distillate 10 g/L 100% ABV) [1]; however, this compound does not carry a specific odor and, therefore, does not contribute to the aroma of the distillates. This compound is formed as a consequence of the degradation of pectins caused by pectolytic enzymes that hydrolyze methoxyl groups during fermentation [18]. It is then transferred to the distillate during the distillation process, and, being very difficult to separate because of its high solubility in water, it becomes present in all the fractions of the distillate [16].

The greater presence of pectolytic enzymes during the fermentation of grape marc explains the high methanol content, being greater than that of other distillates such as brandy [6,19]. During the ageing process, it has been observed that methanol content decreases in WS, WD, and GMD, while no significant differences between the first and second years of ageing could be observed (Table 1). This decrease is attributable to transpiration and evaporation processes, as well as to esterification and oxidation reactions [2,6,9]. On the other hand, an increment of methanol is observed in NA during the first two years of ageing. This slight increase may be caused by the demethylation process caused by lignin, hemicellulose, or xylans, and/or by the contributions from the liquid that had previously been contained in the barrels (brandy in BC and Oloroso sherry wine in SC) [9,20]. The increase in methanol concentration may also be attributed to a phenomenon known as *merma* (transpiration of water through the wood pores), which takes place during ageing [11]. This slight increase in methanol concentration may not be observed in the rest of the distillates as their greater methanol content facilitates to a greater extent the reactions and processes that cause methanol reduction.

Higher alcohols (N-propanol, I-butanol, N-butanol, 2-methyl-1-butanol, 3-methyl-1-butanol, 2-phenylethanol, and 1-hexanol) are the most important volatile compounds from a quantitative point of view. They play a major role in the organoleptic qualities of the distillates and, depending on their concentrations, can even be considered negative [8]. They are formed by yeasts from sugar and amino acids during fermentation [8] and, therefore, both fermentation and distillation conditions are influencing factors with regard to the number of higher alcohols that will be present in the distillates [6,16]. Table S1 shows the individual composition of higher alcohols in the samples analyzed expressed as g/L. The most abundant alcohols in these distillates are isoamyl alcohols (2-methyl-1-butanol and 3-methyl-1-butanol), followed by N-propanol and I-butanol, and, finally, a third group consisting of N-butanol, 2-phenylethanol and 1-hexanol.

**Figure 2:**
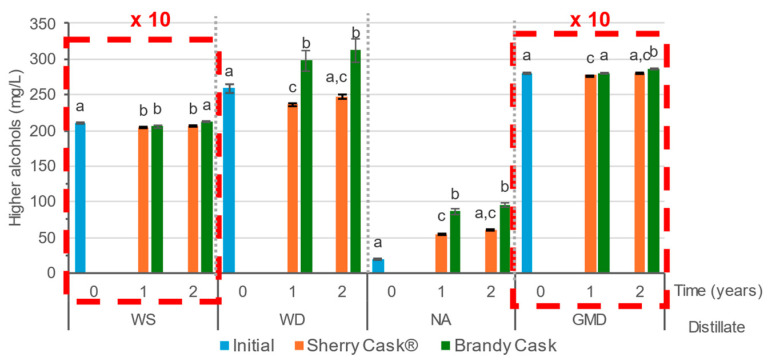
Total higher alcohols in the initial wine spirit (WS), wine distillate (WD), neutral alcohol (NA), and grape marc distillate (GMD) and after ageing for 1 and 2 years. WS and GMD values should be multiplied by a factor of 10. The different letters in a particular distillate indicate significantly different values obtained from the Tukey HSD test (*p* < 0.05).

WS and GMD are the distillates with the highest levels of total higher alcohols, which, as previously noted, are distilled at lower alcoholic strength and the separation of the distillate fractions is not as exhaustive as that achieved with WD or NA (Figure 2). In this study, the total higher alcohol contents in GMD (2810 mg/L) were greater than in WS (2101 mg/L), although in some studies higher alcohol contents in GMD are below those found in some wine distillates [6]. The behavior during ageing does not show a clear trend. The changes are relatively small and, in some cases, without significant differences with the initial distillate. In the case of WS, we can see how total higher alcohols initially decreased during the first year in BC and then they increased to levels similar to those of the initial distillate. The same decrease in total higher alcohols was observed when WS was aged for the first year in SC, after which it remained invariable over the 2nd year. Regarding WD, an increase in total higher alcohols was observed when aged in BC. When aged in SC they decreased slightly, although no significant differences were determined after the second year of ageing with respect to their initial concentration. In the case of NA, which was the distillate with the lowest content of higher alcohols, increases were observed when aged in either cask type, being more pronounced over the first year of ageing than over the second one, and also more noticeable when aged in BC than in SC. Finally, GMD exhibited marginal changes in total higher alcohol content, with a slight increase after 2 years in BC and no significant differences when aged in SC.

These discrepancies between the behavior of the different distillates had already been described in the literature, where we found references to an increment of these compounds during ageing [2,6,11,21] due to their increasing concentration resulting from the evaporation of ethanol (*merma*) and/or by the contribution of the wine or brandy that had been previously contained in the casks. The brandy from the brandy casks made greater contributions of higher alcohols than the sherry from the sherry casks^®^. This could be observed in WD and NA, since brandy has a higher content of these compounds than sherry wine. On the other hand, other authors had observed a decrease [9,22] that was explained by the reactions and processes involving these compounds (esterification, oxidation, evaporation, and sorption).

#### 2.1.3. Polyalcohols

Glycerol is a compound formed by microbiological activity during alcoholic fermentation [8]; therefore, it is present in wine, but not in their distillates due to its high boiling point [12]. Therefore, no glycerol was present in the initial distillates or in the samples aged in BC (Table 1). On the other hand, when the distillates were aged in SCs, the concentration of glycerol increased considerably during ageing as it was contributed by the wine that had been previously contained in the casks [12,23].

2,3-butanediol is a volatile compound that is present in wines through the metabolic pathway of yeasts. It originates from the reduction of the acetoin formed from acetaldehyde [24], which is found in sherry wines, especially in those that undergo biological ageing [13]. In all of the initial distillates, except for GMD, 2,3-butanediol was not present and its evolution during their ageing in BC was nil (Table 1). On the other hand, when aged in SCs, its content increased during their ageing because of the wine seasoning of the cask [12,13].

#### 2.1.4. Esters

Esters are an important group of volatile compounds in distillates, both at qualitative and quantitative levels. They contribute to the organoleptic profile of distillates at appropriate levels [8] and their presence in the distillates is determined by both fermentation and distillation conditions [25]. The main ones are ethyl esters, which are formed during fermentation and increase in concentration during the ageing process of the distillates [9,12]. Ethyl acetate is the major ester that originates from yeast and bacterial metabolism during fermentation, and it is also formed during ageing through the esterification of acetic acid with ethanol [2,11,26]. This behavior was observed in all the distillates aged either in SCs or BCs. GMD presented ethyl acetate concentration at a nearly six times higher level than that of WS or WD, as had already been reported in the literature (Table S2) [6].

**Figure 3:**
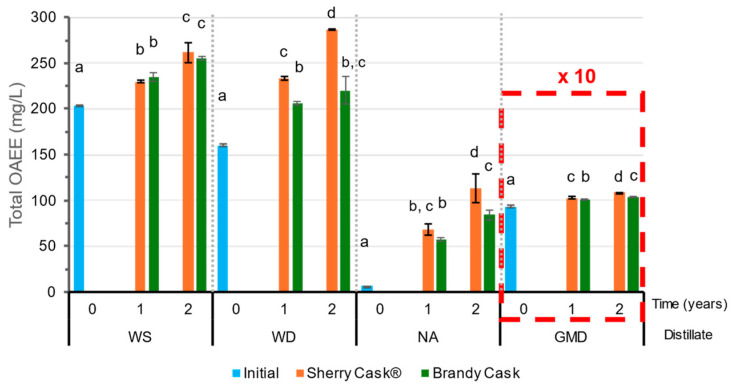
Total organic acids ethyl esters (OAEE) in the initial wine spirit (WS), wine distillate (WD), neutral alcohol (NA), and grape marc distillate (GMD) and after ageing for 1 and 2 years. GMD values should be multiplied by a factor of 10. Different letters indicate that a significant difference has been obtained from the Tukey HSD test (*p* < 0.05).

The total concentration of other esters, such as organic acid ethyl esters (OAEE) (ethyl acetate + ethyl lactate + diethyl malate + diethyl succinate + diethyl tartrate) in the samples studied can be seen in Figure 3, and in greater detail in Table S2. The total OAEE content in GMD was the highest of all the distillates, registered at 930 mg/L in its initial state and reaching 1090 mg/L in the samples aged in SC for 2 years. The second distillate with the highest amount was WS followed by WD. Although WS presented a greater initial concentration, WD exhibited a greater increase during its ageing, mainly due to a more pronounced increment of its ethyl acetate content. NA, in turn, presented the lowest values, with an initial 6.5 mg/L OAEE content mainly attributable to ethyl acetate. This value increased during ageing and reached 84 mg/L and 114 mg/L after two years of ageing in BC and SC, respectively.

Specifically (Table S2), ethyl lactate and diethyl succinate are found at significant levels in the distillates. These compounds contribute to the aromatic complexity and characteristic flavor profile of distilled products. The presence of these esters is a result of the interaction between the organic acids and ethanol during the distillation process [27]. In general, both compounds increase moderately in concentration during ageing. In our study, the distillates aged in SC exhibited a greater increase. This is explained by the fact that both the acids and the esters themselves were present in the wine previously contained in the casks and were in turn transferred to the distillate during the aging process [28]. WS and GMD distillates presented significantly higher concentrations of both compounds (between 50–60 mg/L ethyl lactate and 8–14 mg/L diethyl succinate) compared to those of WD or NA (with 1–8 mg/L ethyl lactate and 1–7 mg/L diethyl succinate).

Diethyl tartrate and diethyl malate were found in the distillates aged in SCs. Both compounds come from the casks’ seasoning wine as well as from the esterification of their respective acids, i.e., tartaric and malic, which also originate from the wine. They presented a lower concentration than the other esters above mentioned, but in all the distillates and for both esters the concentrations increased during ageing and, in certain cases, with a greater increase during the second year of ageing (Table S2).

**Figure 4:**
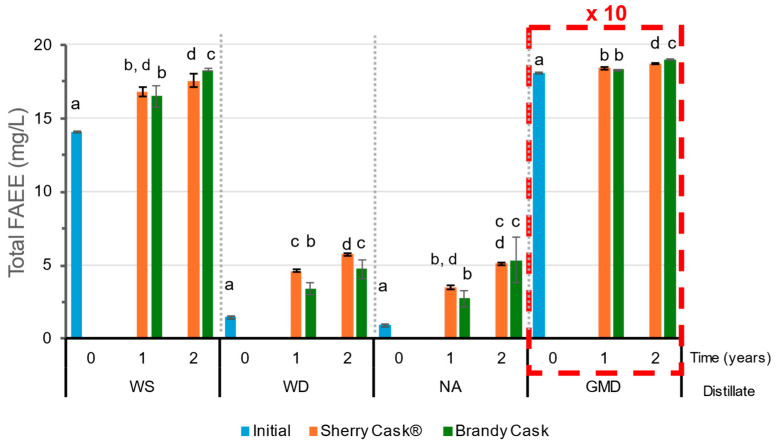
Total initial fatty acid ethyl esters (FAEE) in wine spirit (WS), wine distillate (WD), neutral alcohol (NA), and grape marc distillate (GMD) after ageing for 1 and 2 years. GMD values should be multiplied by a factor of 10. Different letters indicate that a significant difference has been obtained from the Tukey HSD test (*p* < 0.05).

Fatty acid ethyl esters (FAEE), independently of those obtained from the distillation, are also formed by the esterification of ethanol with fatty acids, both during the distillation and during the ageing process [8,25]. These compounds, when found in relatively moderate amounts and particularly in the case of short-chain esters (C6 to C10), contribute positively with fruity and floral notes [8] to the aroma of the final distillates. During the ageing process, some particular esters do not show significant changes while others either increase or decrease slightly; these variations are determined by certain reactions that occur between the compounds present in the medium such as hydrolysis, esterification, or transesterification [9,17], which depend on the type of distillate, although no clear trend has been identified. In general, it was observed that the total sum of fatty acid ethyl esters (FAEE) in all the distillates had increased after the two years of ageing, with slightly higher increases when aged in BC than in SC (except for AN, which did not present any significant variation) (Figure 4). Their contents in the initial WD and NA distillates were very low or even below their quantification limit before the ageing process. Unlike WD and NA, WS exhibited a greater presence of esters at 14 mg/L, which increased up to 18.3 mg/L after two years of ageing in BC. The GMD distillate presented high concentrations of all the esters, with a FAEE value of 181 mg/L that reached 190 mg/L after 2 years of ageing in BC. Most of the esters in both WS and GMD were ethyl octanoate (C8) and ethyl decanoate (C10), followed by ethyl dodecanoate (C12) and ethyl hexadecanoate (C16) (Table S3), which is in agreement with reports from other studies in the literature [5,13].

#### 2.1.5. Acids

**Figure 5:**
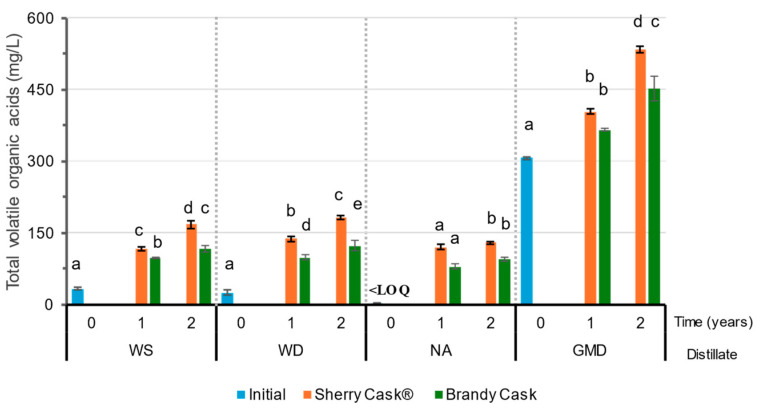
Total initial volatile organic acids in wine spirit (WS), wine distillate (WD), neutral alcohol (NA), and grape marc distillate (GMD) after ageing for 1 and 2 years. A different letter indicates significant differences in a particular row between the values obtained from the Tukey HSD test (*p* < 0.05). LOQ: Limit of quantification.

Volatile acids are important compounds with regard to the aroma of distillates. The major element is acetic acid, which can account for up to 90% of the total acids in the distillate. Its presence in the distillate is determined by the characteristics of the alcoholic fermentation and distillation processes [8]. During ageing, their evolution is marked by several processes, as they are involved in the esterification reactions that form ethyl acetate and increase their concentration both by the oxidation of ethanol and acetaldehyde and by the wood extraction itself, which yields acetic acid through the hydrolysis of the hemicellulose of the wood [2,9,11,13,22]. The concentration values of the volatile organic acids (acetic acid and lactic acid) are given in Table S4, and their total values are represented in Figure 5. It can be observed that WS and WD have similar levels, while NA has a concentration below the LOQ. The concentration of volatile acids in GMD is 10 times higher than that of WS. These differences are explained by the characteristics of the production processes of the distillates. During ageing, a similar behavior was observed in all the distillates, with an increase in acetic acid over the two-year ageing in either BC or SC. In general, the highest levels were obtained in SC after two years (except for NA, where no significant differences could be observed between BC and SC) because of the contributions from the SC seasoning wine [12,28]. Lactic acid was another major volatile acid detected in the distillates, but this one had been formed during alcoholic and/or malolactic fermentation and is a precursor of ethyl lactate [8]. During ageing in BC, a slight increase was observed in all the distillates, which was more noticeable when aged in SC. Given that lactic acid is only found in wood that has been seasoned (SC) and not in plain wood [13], this explains its increased concentration in SC-aged distillates. On the other hand, the increment observed in BC-aged spirits can be explained by its growing concentration due to *merma* and/or by the possible contribution of the brandy that had been previously contained in the casks.

### 2.2. Phenolic and Furfural Compounds

**Figure 6:**
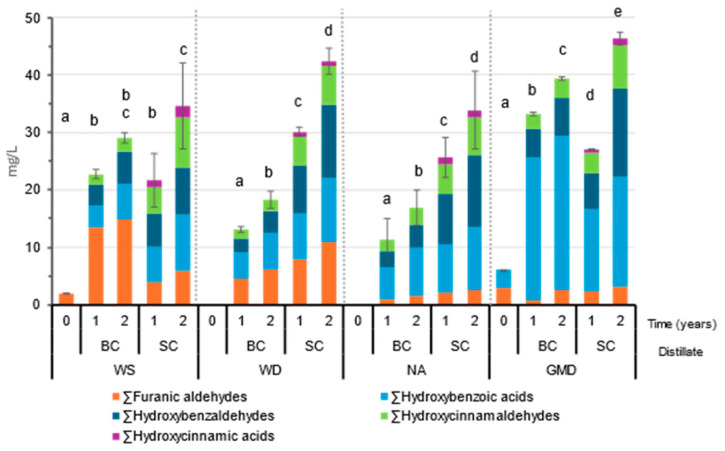
Total initial phenolic compounds and furanic aldehydes in wine spirit (WS), wine distillate (WD), neutral alcohol (NA), and grape marc distillate (GMD) and after ageing for 1 and 2 years. A different letter indicates significant differences in a particular row between the values obtained from the Tukey HSD test (*p* < 0.05).

Phenolic and furfural compounds play an important role in the organoleptic profile of spirits [29]. The global trend of the quantified compounds represented by families can be seen in Figure 6 and in detail in Table S5.

Furanic aldehydes are present in the initial distillates WS and GMD, the concentration of furfural will depend on the conditions of the distillation process [16,27]. During ageing, the concentration of furfural and its derivatives, 5-hydroxymethylfurfural and 5-methylfurfural, increases due to the degradation of the hemicellulose of the wood casks [20]. This increase could be observed in all the distillates, but no clear trend could be identified. For WS and GMD, the increase is greater than for WD and NA, especially in BC, where the concentration was double that obtained in SC. For WD and NA a more moderate increase was observed, which was higher in SC.

The phenolic compounds present in the distillates originated during the ageing process in wood through direct extraction and oxidation reactions [17,22]. The families of aldehydes, hydroxybenzaldehydes (p-hydroxybenzaldehyde, vanillin, and syringaldehyde), and hydroxycinnamaldehydes (coniferaldehyde and sinapaldehyde) presented the expected evolution, with increasing concentration during the ageing process in either BC or SC. These compounds come from the degradation of the wood lignin [20,30]. Vanillin and syringaldehyde were the most important lignin-derived compounds both in terms of concentration and organoleptic properties [2].

The same trend was observed with regard to hydroxybenzoic acids (gallic, ellagic, protocatechuic, vanillic, and syringic) and hydroxycinnamic acids (caffeic and p-coumaric), both of which increased their concentration in all the distillates and particularly in the SC-aged ones. Hydroxybenzoic acids are compounds that come from the oxidation of the aldehydes extracted from the wood lignin (protocatechuic, vanillic, and syringic acids) or from the hydrolysis of tannins (gallic and ellagic acids) [20,30,31]. Hydroxycinnamic acids, on the other hand, come from the SC seasoning wines, which explains why they are only found in SC-aged distillates [20,30,32]. Gallic acid and ellagic acid are not volatile compounds that may play a role in the aromatic profile of the distillates, however, the presence of both of these compounds in the distillates indicates wooden cask ageing and provides certain flavor qualities such as viscosity or texture, particularly in the case of gallic acid [2].

In general, we can determine that phenolic and furfural compounds increased in all the distillates during the ageing process, with SC reaching higher concentrations after 2 years of ageing, with significant differences with respect to BC (except for WS).

This may be due not only to the presence of compounds that come from the seasoning wine, but also to a greater depletion of the casks wood that had been previously used to contain brandy [11].

### 2.3. Multivariate Analysis

A non-directed multivariate analysis was carried out on the quantitative results obtained from the different analyses carried out on the ageing of the distillates. Specifically, a Factorial Analysis using varimax rotation and eigenvalues greater than 1.0 as the selection criterion was conducted with the purpose of determining a small number of factors that explained most of the variability of the 41 variables. Under these conditions, 4 factors were obtained, which altogether could explain 90.1% of the variance.

**Figure 7:**
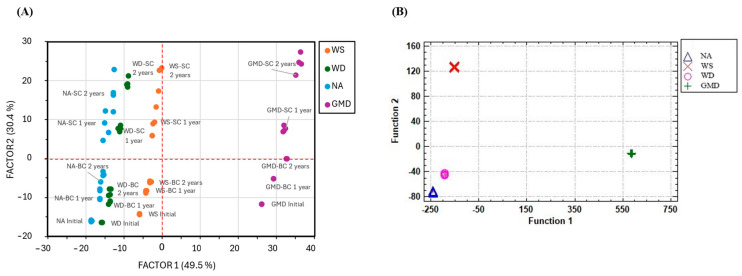
(**A**) Representation of the samples on the plane formed by factors 1 and 2 obtained from the factor analysis. (**B**) Spatial distribution of the functions 1 and 2 obtained through the LDA of the distillates. Wine spirit (WS), wine distillate (WD), neutral alcohol (NA), and grape marc distillate (GMD).

Factors 1 (49.5%) and 2 (30.4%) are represented in Figure 7A, and the coefficients of the factors with r > |0.4| are shown in Table 2. Factor 1 differentiates the samples by distillates, and four groups are identified: GMD, WS, WD, and NA. Factor 2 differentiates the time and conditions of the ageing process. The groups are ordered based on the initial distillate, moving through those aged 1 year in BC, then 2 years in BC, and the furthest away aged in SC.

Table 2 shows that Factor 1 consists of all the volatile compounds found in the distillates at their initial stage (with the exception of 2,3-butanediol), while Factor 2 is made up of compounds that evolved considerably during ageing, such as the phenolic and furfural compounds, i.e., it is their initial volatile composition the one that allows for the initial distillates to be differentiated, while their phenolic composition (phenols and furfurals) the one used to differentiate them after the ageing process. Certain compounds, such as acetic or lactic acids, present a load greater than |0.4| for both factors. This is because these compounds are present in the initial distillates and because their composition evolves during the ageing process due to the physical and chemical reactions that take place over this stage.

Subsequently, a linear discriminant analysis (LDA) was performed on the set of samples in order to generate the equations that would allow an unknown sample to be classified into one of the previously defined groups according to the type of distillate. A total of 80 cases were used to develop a model that discriminates between the four distillate types based on the quantification of the compounds in the samples. A forward stepwise selection method was conducted according to the F-ratio ≥ 4.0 selection criterion. Nineteen variables were determined to be significant predictors for the discrimination of the distillates, and three statistically significant functions were obtained (*p* < 0.05), of which function 1 explains 95.0% of the variation and functions 2 and 3 the remaining 5%.

Thus, Table 3 presents the functions that would allow the classification of the distillate. Among the 19 variables, the most discriminant ones were ethyl hexanoate, ethyl hexadecanoate, ethyl octadecanoate, ethyl lactate, diethyl succinate, diethyl tartrate, 2-phenyl ethanol, benzaldehyde, 1-hexanol, gallic acid, furfural, and p-coumaric acid. A total of 100% of the samples were successfully classified based on the type of distillate (Figure 7B).

### 2.4. Organoleptic Analysis

**Figure 8:**
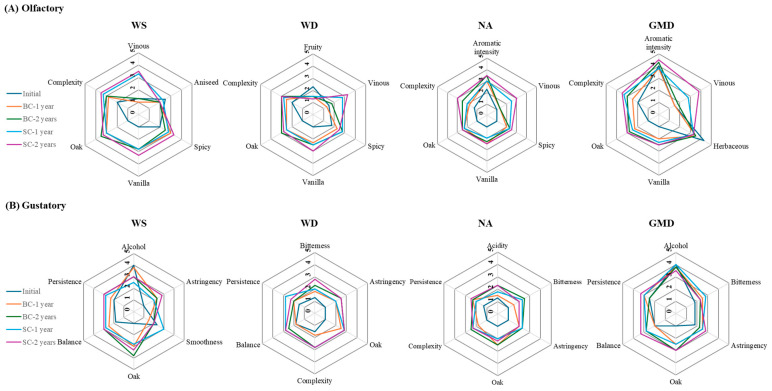
Spider chart comparison of the (**A**) olfactory and (**B**) gustatory profiles of wine spirit (WS), wine distillate (WD), neutral alcohol (NA), and grape marc distillate (GMD) before and after ageing in brandy cask or sherry cask for 1 and 2 years.

Figure 8 shows mean scores granted by the panel to the main olfactory and olfactory-gustatory descriptors of the samples evaluated, and Tables S6 and S7 show the results of all the descriptors after applying an ANOVA to each type of spirit.

The first thing to note is that ageing significantly only affects a few notes in the case of GMD (vanilla and vinous in the nose and oak in the mouth). On the contrary, NA presents the highest number of significant variations among the samples (7 olfactory descriptors (aromatic intensity, vinous, spicy, vanilla, oak, and complexity) and 7 olfactory-gustatory (acidity, bitterness, sweetness, astringency, oak, complexity, balance, and persistence). This is due to the characteristics of the initial NA distillate which presents a neutral profile, as can be observed from the values close to 1.0 for most of the descriptors, which makes any of the changes undergone over the ageing process more conspicuous. In line with this hypothesis, the wine distillate (WD) and the wine spirit (WS) were confirmed to present significant variations regarding the perception of 11 descriptors (4 olfactory (vinous, spicy, vanilla, and oak), 7 gustatory (bitterness, sweetness, astringency, oak, complexity, balance and persistence), 8 descriptors (4 olfactory (vinous, vanilla, oak and complexity), and 4 gustatory (alcohol, astringency, oak and smoothness), respectively.

Some of the descriptors exhibited a repeated behavior in the various distillates studied, as in the case of the olfactory notes of vanilla and oak, which were absent in the initial distillates and appeared at similar intensities after ageing, regardless of both the cask type and the ageing time. The same trend was observed regarding the olfactory-gustatory perception of oak in NA, WD, or GMD, but not in WS, where a greater difference was perceived between the samples according to their ageing time. This allowed us to confirm that the ageing time was a significant factor of variation (*p* = 0.028). On the other hand, the evolution of astringency was similar in NA, WD, and WS, being absent in the initial distillates and present at similar levels (i.e., with a weak/medium intensity) in the different aged samples. On the other hand, a slight astringency was already detected in the initial GMD, which presented no significant differences after the ageing process. It should also be noted that the vinous descriptor was significantly perceived in all of the four distillate types when aged in seasoned casks.

According to the references reviewed, this has been the first time that four types of grape distillates, with very different levels of congeneric compounds and organoleptic characteristics, have been aged in sherry casks or brandy casks at an industrial scale, at the same location, and under similar environmental conditions. The insight resulting from the study of their ageing can contribute to making decisions regarding innovative new products originating either from a single spirit or their blends.

## 3. Materials and Methods

### 3.1. Wooden Casks and Distillate Samples

The distillates used in this study were obtained in accordance with European regulations [1]:

1. WS: Wine spirit distilled at 77% ABV.
2. WD: Wine distillate distilled at 94.7% ABV.
3. NA: Neutral alcohol distilled at 96.0% ABV was obtained through the distillation and rectification of wine.
4. GMD: Grape marc distillate (*Orujo*) distilled at 73% ABV.

All of the distillates were diluted in demineralized water to 68.0% ABV prior to their ageing (Table 4).

This study has been carried out using new 500 L American oak casks (*Quercus alba*) previously subjected to a medium-level toasting procedure, and the following pretreatments:

− SC: The sherry casks^®^ were seasoned for 3 years using 2-year-old Oloroso sherry wine at 18% ABV.
− BC: The brandy casks had been previously used for the ageing of a 65% ABV brandy for 3 years.

The casks were emptied and allowed to fully drain before being filled with the unaged distillates to approximately 485 L.

Both the distillates and the casks were supplied by Bodegas Fundador S.L.U., a winery that belongs to the Protected Geographical Indication “Brandy de Jerez” [4].

**Figure 9:**
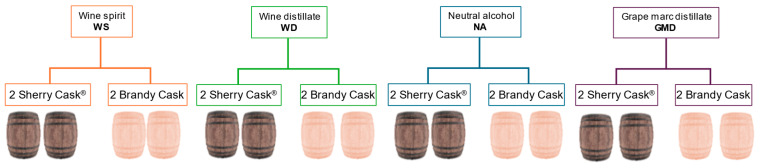
Diagram of the experimental design carried out.

Each experiment was carried out in duplicate, using two casks of each type for each distillate (*n* = 2) (Figure 9). The distillates were statically aged for two years and 700 mL samples were taken to monitor their evolution at 12 and 24 months. The sample volume extracted was not replenished with new distillate. The initial unaged distillates were also analyzed.

### 3.2. Volatile Compounds: Aldehydes, Acetal, Methanol, Higher Alcohols, Esters and Polyalcohols

The methodology described in previous works [11] was used to determine acetaldehyde, acetaldehyde-diethylacetal, benzaldehyde, methanol, higher alcohols, ethyl acetate, fatty acid esters, organic acid esters, glycerol, and 2,3-butanediol. An Agilent 7890B Gas Chromatograph (Agilent Technologies, Santa Clara, CA, USA) coupled to a flame ionization detector was used. The samples were directly injected and analyzed in duplicate. The standards of the determined compounds and the internal standards, 2-pentanol and ethyl undecanoate, were purchased from Sigma Aldrich (Saint Louis, MO, USA). The results were expressed as mg/L.

Total Higher Alcohols were defined as the sum of the concentrations of the following alcohols: [2-butanol] + [N-propanol] + [I-butanol] + [N-butanol] + [2-methyl-1-butanol] + [3-methyl-1-butanol] + [2-phenyl ethanol] + [1-hexanol].

Total fatty acid ethyl esters (FAEE) were defined as the sum of the concentrations of the following esters: [ethyl hexanoate] + [ethyl octanoate] + [ethyl decanoate] + [ethyl dodecanoate] + [ethyl tetradecanoate] + [ethyl hexadecanoate] + [ethyl octadecanoate].

The total acetaldehydes were determined as the sum of the concentrations of acetaldehyde and acetaldehyde-diethylacetal, expressed as acetaldehyde (1 mg diethylacetal is equivalent to 0.373 mg acetaldehyde) [23].

An Acquity UPLC chromatograph (Waters, Milford, MA, USA) equipped with a binary solvent system and an Acquity UPLC^®^ BEH C-18 column (2.1 × 50 mm; 1.7 µm particle size) was used for diethyl tartrate determination. For mass spectroscopy determination, an electrospray ionization (ESI) mass spectrum was used at the positive polarity and registered using a Xevo-G2S Q-TOF (Waters, Milford, MA, USA). The samples and standards were filtered through 0.22 µm nylon filters and injected in duplicate. The calibration line ranged from 0.001 mg/L to 0.5 mg/L. The compounds were identified by comparing their retention times against the mass spectra previously obtained for the standards. The results were expressed as mg/L.

### 3.3. Phenolic Compounds and Furanic Aldehydes

The phenolic compounds and furanic aldehydes were quantified by UHPLC using a Waters Acquity UPLC equipment fitted with a PDA detector and an Acquity UPLC C18 BEH column, 100 × 2.1 mm (i.d.) of 1.7 µm particle size (Waters Corporation, Milford, MA, USA) according to the methodology previously developed [32]. Seven phenolic acids (caffeic acid, p-coumaric acid, ellagic acid, gallic acid, protocatechuic acid, syringic acid, vanillic acid), five phenolic aldehydes (p-hydroxybenzaldehyde, coniferaldehyde, sinapaldehyde, syringaldehyde and vanillin) and three furanic aldehydes (5-hydroxymethylfurfural (5-HMF), 5-methylfurfural and furfural) were quantified.

UHPLC quality acetonitrile and acetic acid (PanReac, Barcelona, Spain), as well as ultrapure water (EMD Millipore, Bedford, MA, USA), were used to prepare the eluents. The standards for calibration were purchased from Sigma Aldrich (Saint Louis, MO, USA).

The samples and standards were filtered through 0.22 µm filters and analyzed in duplicate. The compounds were identified by comparison of their retention times and the UV-Vis spectra of the samples and standards. The calibration curves obtained were within the range of 0.1 mg/L to 20.0 mg/L. The results were expressed as mg/L.

### 3.4. Organic Acids

The organic acids were analyzed using a 930 Compact IC Flex ion chromatography equipment (Metrohm, Madrid, Spain), equipped with a Metrosep Organic Acids column of 250 mm × 7.8 mm (i.d.) and 9 μm particle size. Ultrapure water (EMD Millipore, Bedford, MA, USA), 0.1M sulfuric acid (Sigma-Aldrich, Saint Louis, MO, USA), and UHPLC-quality acetone (VWR International, Radnor, PA, USA) were used for the preparation of the eluents. The acetic, lactic, malic, and succinic acids were purchased from Sigma Aldrich (Saint Louis, MO, USA), and the tartaric acid from PanReac (Barcelona, Spain). The results were expressed as mg/L.

### 3.5. Sensory Analysis

The sensory evaluation sessions were conducted in a room at 22 °C. Thirty mL of each sample were poured into standard black wine glasses [33], which were capped until the time of evaluation to favor the concentration of aromas. The panel consisted of 10 judges, who frequently perform sensory evaluations on this type of sample and are familiar with general tasting procedures.

The samples were analyzed through the standard Descriptive Quantitative Analysis (DQA) [34], using 5-point intensity scales (1: absent; 2: weak; 3: medium; 4: high; 5: very high). The selection of the descriptors was based on the group’s previous works on brandies [25], and was complemented with other descriptors that were more appropriate for marc distillates. Thus, among the olfactory descriptors, the following were analyzed: aromatic intensity, fruity, aldehyde, pungency, vinous, herbaceous, aniseed, spicy, raisin, vanilla, oak, burnt/empyreumatic, complexity; and among the olfactory-gustatory descriptors: alcohol, acidity, bitterness, sweetness, astringency, oak, complexity, smoothness, balance and persistence.

### 3.6. Statistical Analysis

The software package Statgraphics 18 (Statgraphics Technologies, Inc., The Plains, VA, USA) was used for the comparison of means by ANOVA with Tukey’s HSD test (*p* < 0.05) and for the multivariate analysis, i.e., factor analysis (FA) and linear discriminant analysis (LDA). Microsoft^®^ Excel^®^ version 2210 (Microsoft Corp., Redmond, WA, USA) was used for the rest of the statistical parameters and graphs.

The Shapiro–Wilk test (*p* < 0.05) was used to verify the normality of the sensory data for each descriptor in each group. The Leven test was used to confirm the equality of variances between the groups compared. Once the requirements of the parametric statistics were met, the analysis of variance was applied to investigate the sensory differences between the groups of samples considered. The analysis of variance and the factor analysis of the results from the sensory analysis were carried out using Statistica 7.0 (StatSoft. Inc., Tulsa, OK, USA).

## 4. Conclusions

This study has demonstrated that the ageing process in oak casks: sherry cask^®^ or brandy cask, exerts a significant influence on the chemical composition and sensory profile of different distillates derived from grapes. 

The changes in the volatile composition of the aged distillates were marked by the type of cask used, with greater increases of such composition in the distillates aged in SC, mainly concerning volatile organic acids and organic acid esters, as well as compounds contributed by the wine used during the cask seasoning process (organic acids and their corresponding ethyl esters, glycerol and 2,3-butanediol). Phenolic and furfural compounds increased considerably in either cask type over the ageing process.

The differences between the different distillates were already reflected in the FA performed, where the samples analyzed were distributed in the space formed by the first two factors; the type of distillate (Factor 1) and the type of ageing (Factor 2), more specifically the type of cask used and the ageing time.

These differences were clearly reflected by the result obtained when applying an LDA to the samples analyzed, which showed a 100% discrimination between the 4 distillates. This discrimination was mainly related to fatty acid ethyl esters and some higher alcohols, such as acetic acid, ethyl acetate and total aldehydes, among others. This again corroborates the relevance of the initial distillate composition with regard to the final product to be obtained.

The sensory analysis confirmed that ageing significantly modifies the organoleptic characteristics of the distillates in comparison with unaged distillates, with neutral alcohol showing the greatest number of significant variations among the samples, while grape marc distillate exhibited the fewest number of descriptors that presented significant differences after completing its ageing process.

The results obtained provide valuable information for the industry, as they allow a better understanding of the processes involved and facilitate the production of spirits with specific organoleptic characteristics, which represents an opportunity for innovation and for the development of new products.

## Figures and Tables

**Table 1:** Initial benzaldehyde, methanol, glycerol, and 2,3-butanediol (mg/L) in wine spirit (WS), wine distillate (WD), neutral alcohol (NA), and grape marc distillate (GMD) and after ageing for 1 and 2 years.

			Brandy Cask (BC)		Sherry Cask (SC)	
Time (Years)		0	1	2	1	2
Benzaldehyde	WS	<LOQ	0.19 ± 0.02 ^a^	0.18 ± 0.01 ^a^	0.20 ± 0.04 ^a^	0.20 ± 0.03 ^a^
	WD	<LOQ	0.12 ± 0.02 ^a^	0.11 ± 0.01 ^a^	0.17 ± 0.02 ^b^	0.23 ± 0.02 ^c^
	NA	<LOQ	0.41 ± 0.05 ^a^	0.67 ± 0.08 ^b^	0.16 ± 0.01 ^c^	0.49 ± 0.01 ^a^
	GMD	3.75 ± 0.19 ^a^	3.20 ± 0.04 ^b^	3.23 ± 0.02 ^b^	3.69 ± 0.05 ^a^	3.97 ± 0.04 ^c^
Methanol	WS	449.52 ± 0.41 ^a^	408.79 ± 9.61 ^b,c^	404.59 ± 4.52 ^b^	424.07 ± 1.75 ^d^	418.22 ± 5.49 ^c,d^
	WD	472.66 ± 10.49 ^a^	451.17 ± 5.00 ^b^	440.43 ± 6.93 ^b,c^	433.94 ± 1.41 ^c,d^	424.01 ± 4.9 ^d^
	NA	25.19 ± 0.51 ^a^	39.56 ± 0.98 ^b^	42.88 ± 0.40 ^c^	32.70 ± 1.08 ^d^	36.49 ± 2.00 e
	GMD	1380.77 ± 4.99 ^a^	1343.39 ± 6.81 ^b^	1313.11 ± 8.16 ^c^	1348.31 ± 8.68 ^b^	1326.00 ± 0.81 ^c^
Glycerol	WS	N.D.	N.D.	N.D.	182.87 ± 15.41 ^a^	210.16 ± 10.78 ^a^
	WD	N.D.	N.D.	N.D.	169.31 ± 10.4 ^a^	351.7 ± 51.46 ^b^
	NA	N.D.	N.D.	N.D.	124.57 ± 10.59 ^a^	176.49 ± 4.34 ^b^
	GMD	N.D.	N.D.	N.D.	179.35 ± 2.41 ^a^	259.9 ± 4.08 ^b^
2,3-Butanediol	WS	N.D.	N.D.	N.D.	18.66 ± 0.84 ^a^	22.07 ± 0.79 ^b^
	WD	N.D.	N.D.	N.D.	25.18 ± 0.61 ^a^	31.23 ± 0.52 ^b^
	NA	N.D.	N.D.	N.D.	21.72 ± 1.65 ^a^	24.84 ± 0.27 ^b^
	GMD	16.44 ± 0.31 ^a^	15.59 ± 0.07 ^a^	16.41 ± 0.01 ^a^	40.89 ± 0.41 ^b^	45.68 ± 0.69 ^c^

Mean ± standard deviation (*n* = 4); a different letter indicates significant differences in a particular row between the values obtained from the Tukey HSD test (*p* < 0.05). N.D., non-detected. LOQ, limit of quantification.

**Table 2:** Factor 1 and Factor 2 where r > |0.4| for the factor analysis performed with the major volatile compounds.

	Factor 1	Factor 2		Factor 1	Factor 2
Total acetaldehyde	0.99		Benzaldehyde	0.98	
Methanol	0.95		1-Hexanol	0.98	
n-Propanol	0.77		Diethyl malate		0.68
Ethyl acetate	0.99		Gallic acid		0.53
I-Butanol	0.89		5-HMF		0.78
n-Butanol	0.88		Protocatechuic acid		0.85
2-Methyl-1-butanol	0.75		Furfural	0.68	
3-Methyl-1-butanol	0.64		Vanillic acid		0.56
Ethyl hexanoate	0.99		p-Hydroxybenzaldehyde		0.41
Ethyl octanoate	0.99		5-Methylfrufural		0.75
Ethyl decanoate	0.99		Syringic acid		0.86
Ethyl dodecanoate	0.99		Vanillin		0.62
Ethyl tetradecanoate	0.99		Caffeic acid		0.90
Ethyl hexadecanoate	0.99		p-Coumaric acid		0.92
Ethyl octadecanoate	0.73	0.43	Syringaldehyde		0.87
Glycerol		0.95	Coniferaldehyde		0.80
Ethyl Lactate	0.63		Sinapaldehyde		0.87
Diethyl Succinate	0.42	0.58	Ellagic acid		0.81
Diethyl Tartrate		0.91	Lactic acid	0.53	0.75
2-Phenyl ethanol	0.92		Acetic acid	0.92	
2,3-Butanediol	0.54	0.82			

**Table 3:** Coefficients of classification functions for wine spirit, wine distillate, neutral alcohol, and grape marc distillate.

	WS	WD	NA	GMD
Total acetaldehyde	21.0	25.6	2.0	215.5
N-Propanol	51.2	59.1	2.6	124.1
Ethyl acetate	−27.8	−14.4	−1.4	−116.7
I-Butanol	16.4	−24.4	−0.2	89.2
n-Butanol	377.2	146.9	6.4	−66.2
3-Methyl-1-butanol	5.9	−5.4	−0.3	−36.2
Ethyl hexanoate	−893.7	−240.9	−16.9	−2888.1
Ethyl hexadecanoate	49.1	425.5	33.6	3539.2
Ethyl octadecanoate	723.2	496.1	−36.1	−2950.9
Ethyl lactate	88.3	38.1	−1.3	−525.7
Diethyl succinate	197.2	114.6	24.7	2040.2
Diethyl tartrate	267.7	93.2	−6.6	576.0
2-Phenyl ethanol	−188.9	−240.8	−9.8	−1253.2
Benzaldehyde	−460.2	159.2	42.5	3419.9
1-Hexanol	574.9	259.9	37.3	6051.3
Gallic acid	−38.2	−84.5	−2.5	−293.3
Furfural	61.3	39.4	6.9	718.8
p-Coumaric acid	−106.8	368.3	16.5	996.9
Acetic acid	4.0	4.1	0.2	7.5
Constant	−18,485.9	−5297.2	−37.6	−316,687.0

**Table 4:** Alcoholic strength and pH of the initial wine spirit (WS), wine distillate (WD), neutral alcohol (NA), and grape marc distillate (GMD).

	Alcoholic Strength (%ABV)		
	As Distilled	Diluted for Ageing	pH
WS	77.07 ± 0.01	68.67 ± 0.01	5.01 ± 0.01
WD	94.70 ± 0.02	68.40 ± 0.01	4.62 ± 0.01
NA	96.02 ± 0.01	68.67 ± 0.00	6.33 ± 0.01
GMD	73.06 ± 0.03	68.82 ± 0.01	4.24 ± 0.02

## Data Availability

Data are contained within the article and Supplementary Materials.

## Supplementary Materials

The following supporting information can be downloaded at: https://www.mdpi.com/article/10.3390/molecules29225303/s1, Table S1. Higher alcohols (mg/L) in wine spirit (WS), wine distillate (WD), neutral alcohol (NA) and grape marc distillate (GMD) initials and after ageing for 1 y 2 years. Table S2. Organic acid ethyl esters (mg/L) in wine spirit (WS), wine distillate (WD), neutral alcohol (NA), and grape marc distillate (GMD) initials and after ageing for 1 y 2 years. Table S3. Fatty acid ethyl esters (mg/L) in wine spirit (WS), wine distillate (WD), neutral alcohol (NA), and grape marc distillate (GMD) initials and after ageing for 1 y 2 years. Table S4. Volatile organic acids (mg/L) in wine spirit (WS), wine distillate (WD), neutral alcohol (NA), and grape marc distillate (GMD) initials and after ageing for 1 y 2 years. Table S5. Phenolic compounds and furfural aldehydes (mg/L) in wine spirit (WS), wine distillate (WD), neutral alcohol (NA), and grape marc distillate (GMD) initials and after ageing for 1 y 2 years. Table S6. Scores on the olfactory sensory descriptors of the samples and analysis of variance of the data for each type of spirit. Table S7. Scores on the olfactory-gustatory sensory descriptors of the samples and analysis of variance of the data for each type of spirit.
