# Supplementary material for: Chemical and Sensory Profile of Grape Distillates Aged in Quercus alba Casks Previously Used for Sherry Wine or Brandy
Source: Molecules. 2024 Nov 9;29(22):5303. doi: 10.3390/molecules29225303 (PMC11596441; doi:10.3390/molecules29225303)
Supplement: Supplementary file 1 [file molecules-29-05303-s001.zip › molecules-3260605-supplementary.pdf]

Supplementary Materials

Chemical and Sensory Profile of Grape Distillates Aged in *Quercus alba* Casks Previously Used for Sherry Wine or Brandy

Daniel Butrón-Benítez [1,2], Manuel J. Valcárcel-Muñoz [3], M. Valme García-Moreno [1,*], M. Carmen Rodríguez-Dodero [1] and Dominico A. Guillén-Sánchez [1,*]

[1] Departamento de Química Analítica, Facultad de Ciencias, Instituto Investigación Vitivinícola y Agroalimentaria (IVAGRO), Universidad de Cádiz, Campus Universitario de Puerto Real, 11510 Puerto Real, Cádiz, Spain.

[2] Bodegas Fundador S.L.U, C/ San Ildefonso, nº 3, 11403 Jerez de la Frontera, Cádiz, Spain.

[3] Oeno R&D FL, 11402 Jerez de la Frontera, Cádiz, Spain

* Correspondence: valme.garcia@uca.es; dominico.guillen@uca.es

Table S1. Higher alcohols (mg/L) in wine spirit (WS), wine distillate (WD), neutral alcohol (NA) and grape marc distillate (GMD) initials and after ageing for 1 y 2 years.

			**Brandy Cask (BC)**		**Sherry Cask (SC)**	
**Time (years)**		**0**	**1**	**2**	**1**	**2**
N-propanol	WS	225.50±0.94 [a]	219.58±3.24 [b]	226.43±1.16 [a]	214.32±1.48 [c]	216.01±1.14 [b,c]
	WD	150.55±0.46 [a]	150.52±0.93 [a]	153.40±0.61 [b]	142.06±1.50 [c]	146.52±1.80 [d]
	NA	5.30±0.17 [a]	15.57±3.88 [b]	16.92±3.57 [b]	7.68±0.54 [a]	8.09±0.08 [a]
	GMD	324.31±1.21 [a]	326.06±0.14 [a]	324.33±0.90 [a]	328.36±1.11 [a]	335.79±0.04 [b]
I-butanol	WS	236.28±0.21 [a]	235.36±4.65 [a]	243.42±3.49 [b]	228.21±1.32 [c]	230.52±2.24 [a, c]
	WD	33.28±1.66 [a]	39.02±1.64 [b]	40.50±3.03 [b]	32.17±0.83 [a]	33.61±1.00 [a]
	NA	3.01±0.49 [a]	13.54±2.51 [b]	14.80±2.43 [b]	6.81±0.27 [c]	7.63±0.45 [c]
	GMD	493.06±0.36 [a,b]	493.53±1.21 [a]	503.13±2.45 [c]	487.10±0.80 [b]	504.47±1.78 [c]
N-butanol	WS	9.98±1.31 [a]	10.15±0.59 [a]	10.42±0.19 [a]	9.99±0.24 [a]	10.03±0.33 [a]
	WD	N.D.	N.D.	N.D.	N.D.	N.D.
	NA	N.D.	N.D.	N.D.	N.D.	N.D.
	GMD	21.61±0.47 [a]	21.81±0.11 [a]	21.77±0.37 [a]	21.19±0.53 [a,b]	20.79±0.07 [b]
2-methyl-1-butanol	WS	285.90±0.03 [a]	270.18±4.60 [b]	282.80±0.85 [a]	271.59±1.75 [b]	275.07±0.86 [b]
	WD	13.16±2.08 [a]	19.23±2.40 [b]	20.77±2.85 [b]	10.94±0.08 [a]	12.00±0.12 [a]
	NA	1.74±0.02 [a]	9.77±0.29 [b]	10.69±0.44 [c]	6.75±0.10 [d]	7.38±0.28 [e]
	GMD	392.09±1.44 [a]	389.09±0.71 [a]	396.61±1.82 [b]	380.83±0.35 [c]	387.45±0.11 [a]
3-methyl-1-butanol	WS	1326.75±4.75 [a]	1301.54±16.81 [b,c]	1350.19±4.12 [d]	1290.67±6.25 [b]	1310.89±7.56 [a, c]
	WD	60.66±2.55 [a]	87.47±10.45 [b]	95.44±9.16 [b]	46.54±0.38 [c]	50.14±0.48 [a, c]
	NA	8.11±0.12 [a]	45.42±1.71 [b]	51.34±2.06 [c]	29.87±0.26 [d]	32.50±0.30 [d]
	GMD	1491.11±1.59 [a]	1491.88±3.37 [a]	1526.04±7.80 [b]	1463.23±1.46 [c]	1467.17±1.19 [c]
2-phenylethanol	WS	5.89±0.14 [a]	4.66±0.25 [b]	4.04±0.53 [b]	9.39±0.21 [c]	10.10±0.43 [c]
	WD	0.28±0.07 [a]	0.56±0.18 [a]	0.69±0.24 [a]	3.83±0.21 [b]	4.81±0.25 [c]
	NA	0.27±0.11 [a]	0.46±0.12 [a,b]	0.73±0.06 [b]	3.56±0.37 [c]	4.14±0.00 [d]
	GMD	17.69±0.28 [a,b]	17.53±0.25 [a]	18.38±0.03 [b]	22.67±0.08 [c]	23.84±0.25 [d]
1-hexanol	WS	11.30±0.18 [a]	10.55±0.28 [b]	10.01±0.07 [c]	10.52±0.14 [b]	10.66±0.22 [b]
	WD	0.67±0.01 [a,b]	0.94±0.20 [b]	1.26±0.20 [c]	0.50±0.05 [a]	0.51±0.03 [a]
	NA	0.13±0.01 [a]	0.49±0.09 [b,c]	0.55±0.14 [b]	0.37±0.01 [c, d]	0.22±0.02 [a, d]
	GMD	70.95±0.22 [a]	69.60±0.45 [b]	70.15±0.02 [b]	70.30±0.62 [b]	70.37±0.10 [b]
**Total Higher alcohols**	WS	2101.61±4.10 [a]	2052.03±28.04 [b]	2127.31±5.45 [a]	2034.70±9.45 [b]	2063.28±11.59 [b]
	WD	258.60±5.78 [a]	297.75±14.87 [b]	312.06±16.02 [b]	236.04±2.66 [c]	247.59±3.58 [a, c]
	NA	19.75±0.28 [a]	86.56±4.00 [b]	95.99±3.35 [c]	55.05±0.63 [d]	59.97±0.85 [d]
	GMD	2810.82±1.96 [a]	2809.50±1.68 [a]	2860.41±13.33 [b]	2773.68±4.10 [c]	2809.89±2.62 [a]

Mean ± standard deviation (n=4); A different letter indicates significant differences in a particular row between the values obtained from the Tukey HSD test ($p < 0.05$). N.D., non-detected. LOQ, limit of quantification.

Table S2. Organic acid ethyl esters (mg/L) in wine spirit (WS), wine distillate (WD), neutral alcohol (NA) and grape marc distillate (GMD) initials and after ageing for 1 y 2 years.

			**Brandy Cask (BC)**		**Sherry Cask (SC)**	
**Time (years)**		**0**	**1**	**2**	**1**	**2**
**Ethyl acetate**	WS	147.76±2.85 [a]	179.36±5.56 [b]	200.58±1.38 [c]	160.76±1.89 [d]	187.29±9.35 [b]
	WD	157.66±1.55 [a]	200.39±3.24 [b]	214.19±12.94 [c]	217.95±2.27 [c]	251.96±0.59 [d]
	NA	3.66±0.12 [a]	51.69±0.87 [b]	76.52±3.75 [c]	57.87±6.30 [b]	91.69±15.23 [c]
	GMD	872.83±13.63 [a]	956.22±6.62 [b]	981.74±7.05 [c]	954.67±15.06 [b]	1011.05±0.53 [d]
**Ethyl lactate**	WS	47.38±1.98 [a]	49.19±0.74 [a,b]	47.36±1.43 [a]	49.19±0.74 [a,b]	50.80±1.35 [b]
	WD	2.05±0.17 [a]	5.06±1.50 [b]	5.26±1.72 [b]	6.59±0.20 [b,c]	8.49±0.32 [c]
	NA	0.82±0.00 [a]	4.35±1.10 [b]	6.04±1.27 [c]	4.82±0.01 [b,c]	6.35±0.02 [c]
	GMD	50.22±0.47 [a]	52.19±0.19 [b]	54.40±0.02 [c]	54.96±0.08 [c]	57.43±0.54 [d]
**Diethyl malate**	WS	N.D.	N.D.	N.D.	2.16±0.11	2.87±0.13
	WD	N.D.	N.D.	N.D.	0.96±0.22	14.88±0.72
	NA	N.D.	N.D.	N.D.	0.83±0.10	8.22±0.56
	GMD	N.D.	N.D.	N.D.	2.07±0.04	3.58±0.03
**Diethyl succinate**	WS	8.21±0.35 [a]	6.57±0.28 [b]	7.22±0.10 [b]	12.49±0.34 [c]	14.28±0.46 [d]
	WD	0.48±0.06 [a]	0.57±0.11 [a]	0.68±0.11 [a]	4.70±0.10 [b]	6.99±0.11 [c]
	NA	<LOQ	<LOQ	<LOQ	3.49±0.39 [a]	5.15±0.01 [b]
	GMD	7.25±0.08 [a]	7.00±0.08 [a]	7.39±0.02 [a]	11.00±0.02 [b]	12.67±0.10 [c]
**Diethyl tartrate**	WS	N.D.	N.D.	N.D.	5.32±0.20 [a]	6.20±0.27 [b]
	WD	N.D.	N.D.	N.D.	2.90±0.13 [a]	4.20±0.29 [b]
	NA	N.D.	N.D.	N.D.	1.45±0.22 [a]	2.17±0.35 [b]
	GMD	N.D.	N.D.	N.D.	3.34±0.20 [a]	5.42±0.02 [b]
**∑OAEE**	WS	203.35±0.51 [a]	235.12±5.03 [b]	255.16±2.34 [c]	229.92±1.80 [b]	261.44±10.57 [c]
	WD	160.18±1.32 [a]	206.02±2.44 [b]	220.13±14.77 [b,c]	233.10±1.93 [c]	286.51±0.82 [d]
	NA	6.52±0.12 [a]	57.44±1.91 [b]	84.32±5.27 [c]	68.46±6.57 [b,c]	113.57±15.58 [d]
	GMD	930.30±14.02 [a]	1015.41±6.89 [b]	1043.52±7.05 [c]	1026.04±14.84 [c]	1090.16±0.12 [d]

Mean ± standard deviation (n=4); A different letter indicates significant differences in a particular row between the values obtained from the Tukey HSD test ($p < 0.05$). N.D., non-detected. LOQ, limit of quantification.

Table S3. Fatty acid ethyl esters (mg/L) in wine spirit (WS), wine distillate (WD), neutral alcohol (NA) and grape marc distillate (GMD) initials and after ageing for 1 y 2 years.

			**Brandy Cask (BC)**		**Sherry Cask (SC)**	
**Time (years)**		**0**	**1**	**2**	**1**	**2**
SHORT-CHAIN FAEE						
Ethyl hexanoate	WS	1.49±0.09 ^{a}	<LOQ	<LOQ	1.65±0.05 ^{b}	1.85±0.05 ^{c}
	WD	N.D.	N.D.	N.D.	N.D.	N.D.
	NA	N.D.	N.D.	N.D.	0.10±.0.00 ^{a}	0.20±0.02 ^{b}
	GMD	17.90±0.58 ^{a}	17.01±0.27 ^{b,c}	17.39±0.07 ^{a,b}	16.71±0.22 ^{c}	16.91±0.16 ^{c}
Ethyl octanoate	WS	5.61±0.05 ^{a}	7.43±0.30 ^{b}	8.22±0.51 ^{c}	6.49±0.05 ^{d}	6.74±0.21 ^{d}
	WD	0.36±0.01 ^{a}	1.25±0.15 ^{b}	1.95±0.29 ^{c}	1.87±0.04 ^{c}	2.13±0.09 ^{c}
	NA	<LOQ	0.88±0.09 ^{a,b}	2.28±0.46 ^{c}	0.72±0.10 ^{a}	1.33±0.10 ^{b}
	GMD	44.53±0.12 ^{a}	46.26±0.22 ^{b}	47.12±0.05 ^{c}	46.80±0.39 ^{b,c}	46.77±0.23 ^{c}
Ethyl decanoate	WS	4.57±0.03 ^{a}	5.75±0.14 ^{b}	5.75±0.05 ^{b}	5.52±0.20 ^{b}	5.63±0.19 ^{b}
	WD	0.57±0.04 ^{a}	1.22±0.17 ^{b}	1.42±0.27 ^{b}	1.21±0.07 ^{b}	1.48±0.03 ^{b}
	NA	0.33±0.01 ^{a}	0.93±0.31 ^{a,b}	1.32±0.93 ^{b}	1.20±0.02 ^{a,b}	1.66±0.01 ^{b}
	GMD	63.40±0.12 ^{a}	63.83±0.07 ^{a}	65.02±0.04 ^{b}	63.57±0.18 ^{a}	63.88±0.08 ^{a}
LON-CHAIN FAEE						
ethyl dodecanoate	WS	0.84±0.03 ^{a}	1.43±0.08 ^{b}	1.69±0.18 ^{c}	1.16±0.04 ^{d}	1.19±0.04 ^{d}
	WD	0.31±0.05 ^{a}	0.56±0.12 ^{b}	0.72±0.13 ^{b}	0.64±0.04 ^{b}	0.74±0.01 ^{b}
	NA	0.26±0.00 ^{a}	0.55±0.17 $^{a, b,c}$	0.83±0.25 ^{c}	0.50±0.04 ^{a,b}	0.64±0.02 ^{b,c}
	GMD	26.47±0.04 ^{a}	26.97±0.04 ^{b}	28.15±0.01 ^{c}	26.74±0.03 ^{d}	27.35±0.01 e
ethyl tetradecanoate	WS	0.25±0.06 ^{a}	0.44±0.02 ^{b}	0.55±0.06 ^{c}	0.25±0.03 ^{a}	0.27±0.03 ^{a}
	WD	0.11±0.00 ^{a}	0.11±0.01 ^{a}	0.14±0.01 ^{b}	0.09±0.01 ^{a}	0.13±0.01 ^{b}
	NA	0.14±0.03 ^{a,b}	0.13±0.01 ^{a}	0.18±0.01 ^{b}	0.16±0.04 ^{a,b}	0.08±0.00 ^{c}
	GMD	7.18±.0.00 ^{a}	7.31±0.09 ^{b}	7.70±0.06 ^{c}	7.28±0.02 ^{a,b}	7.40±0.01 ^{b}
ethyl hexadecanoate	WS	0.90±0.15 ^{a}	1.25±0.35 ^{a}	1.78±0.27 ^{b}	1.20±0.12 ^{a}	1.32±0.10 ^{a,b}
	WD	0.12±0.01 ^{a}	0.27±0.02 ^{b}	0.50±0.04 ^{c}	0.48±0.02 ^{c}	0.76±0.02 ^{d}
	NA	0.11±0.03 ^{a}	0.26±0.03 ^{b}	0.73±0.10 ^{c}	0.53±0.03 ^{d}	0.65±0.00 ^{c}
	GMD	20.85±0.25 ^{a}	20.99±0.37 ^{a}	23.28±0.08 ^{b}	22.14±0.03 ^{c}	23.45±0.02 ^{b}
ethyl octadecanoate	WS	0.39±0.02 ^{a}	0.18±0.02 ^{b}	0.28±0.03 ^{b}	0.52±0.03 ^{c}	0.54±0.10 ^{c}
	WD	<LOQ	<LOQ	<LOQ	0.32±0.02 ^{a}	0.51±0.03 ^{b}
	NA	<LOQ	<LOQ	<LOQ	0.32±0.01 ^{a}	0.57±0.02 ^{b}
	GMD	0.66±0.11 ^{a,b}	0.56±0.02 ^{b}	1.07±0.03 ^{c}	0.65±0.03 ^{b}	0.77±0.01 ^{b}
**∑FAEE**	WS	14.05±0.00 ^{a}	16.48±0.71 ^{b}	18.26±0.11 ^{c}	16.79±0.33 $^{b, d}$	17.53±0.44 ^{d}
	WD	1.47±0.08 ^{a}	3.40±0.39 ^{b}	4.73±0.66 ^{c}	4.61±0.08 ^{c}	5.75±0.10 ^{d}
	NA	0.92±0.06 ^{a}	2.75±0.54 ^{b}	5.34±1.54 ^{c}	3.52±0.15 $^{b, d}$	5.13±0.09 $^{c, d}$
	GMD	180.98±0.06 ^{a}	182.94±0.07 ^{b}	189.72±0.11 ^{c}	183.89±0.75 ^{b}	186.53±0.48 ^{d}

Mean ± standard deviation (n=4); A different letter indicates significant differences in a particular row between the values obtained from the Tukey HSD test ($p < 0.05$). N.D., non-detected. LOQ, limit of quantification.

Table S4. Volatile organic acids (mg/L) in wine spirit (WS), wine distillate (WD), neutral alcohol (NA) and grape marc distillate (GMD) initials and after ageing for 1 y 2 years.

			Brandy Cask (BC)		Sherry Cask (SC)	
Time (years)		0	1	2	1	2
**Acetic acid**	WS	30.28±2.63 [a]	93.32±1.93 [b]	111.98±6.87 [c]	106.10±7.53 [b,c]	156.89±9.39 [d]
	WD	24.69±5.28 [a]	94.69±4.90 [b]	120.45±10.89 [c]	130.07±1.99 [c]	173.64±4.55 [d]
	NA	<LOQ	74.97±5.32 [a]	114.52±9.15 [b]	88.30±13.21 [a]	121.69±13.46 [b]
	GMD	300.85±2.08 [a]	359.25±3.59 [b]	445.20±26.40 [c]	405.15±2.19 [b]	527.08±11.35 [d]
**Lactic acid**	WS	2.16±0.09 [a]	4.68±0.50 [b]	4.98±0.22 [b]	9.01±0.36 [c]	9.94±0.41 [d]
	WD	<LOQ	3.05±0.46 [a]	2.56±0.67 [a]	7.09±0.29 [b]	8.61±0.28 [c]
	NA	1.87±0.86 [a]	4.12±0.63 [b]	4.67±0.60 [b]	6.15±0.38 [c]	7.27±0.53 [c]
	GMD	5.77±0.76 [a]	6.29±0.16 [a]	6.29±0.16 [a]	12.85±2.01 [b]	19.04±0.87 [c]

Mean ± standard deviation (n=4); A different letter indicates significant differences in a particular row between the values obtained from the Tukey HSD test (p< 0.05). N.D., non-detected. LOQ, limit of quantification.

Table S5. Phenolic compounds and furfural aldehydes (mg/L) in wine spirit (WS), wine distillate (WD), neutral alcohol (NA) and grape marc distillate (GMD) initials and after ageing for 1 y 2 years.

			**Brandy Cask (BC)**		**Sherry Cask (SC)**	
**Time (years)**		**0**	**1**	**2**	**1**	**2**
Furfural	WS	1.92±0.07 [a]	12.77±0.39 [b]	14.13±0.05 [c]	2.99±0.37 [d]	4.25±0.71 [e]
	WD	<LOQ	3.05±0.20 [a]	3.50±0.32 [a]	2.10±0.39 [a]	3.06±0.67 [b]
	NA	N.D.	3.00±1.10 [a]	3.63±1.26 [b]	1.88±0.90 [a,b]	2.42±0.86 [c]
	GMD	6.07±0.00 [a]	22.83±0.22 [c]	24.31±0.29 [c]	11.54±0.26 [b]	12.73±0.28 [b]
5-HMF	WS	N.D.	0.56±0.14 [a]	0.56±0.10 [a]	0.73±0.21 [a,b]	1.04±0.36 [b]
	WD	N.D.	0.41±0.02 [a]	0.47±0.03 [a]	0.86±0.08 [b]	1.15±0.21 [c]
	NA	N.D.	0.54±0.26 [a,b]	0.70±0.38 [a,b]	0.82±0.44 [b]	1.03±0.47 [b]
	GMD	N.D.	0.60±0.03 [a]	0.73±0.06 [a]	0.76±0.00 [a]	1.74±0.05 [b]
5-methylfurfural	WS	N.D.	0.15±0.02 [a]	0.16±0.01 [a]	0.30±0.14 [a]	0.63±0.12 [b]
	WD	N.D.	0.11±0.03 [a]	0.14±0.06 [a]	0.29±0.07 [b]	0.38±0.08 [b]
	NA	N.D.	0.14±0.08 [a]	0.19±0.04 [a]	0.12±0.09 [a]	0.15±0.10 [a]
	GMD	N.D.	<LOQ	0.22±0.00 [a]	0.21±0.01 [a]	0.59±0.11 [b]
∑Furanic aldehydes	WS	1.92±0.07 [a]	13.48±0.36 [b]	14.85±0.05 [c]	4.03±0.44 [d]	5.92±0.95 [e]
	WD	<LOQ	0.74±0.05 [a]	1.04±0.19 [a]	2.17±0.27 [b]	2.59±0.27 [b]
	NA	N.D.	1.00±0.21 [a]	1.57±0.45 [a. b]	2.13±0.10 [b. c]	2.50±0.36 [c]
	GMD	6.07±0.00 [a]	23.44±0.25 [b]	25.26±0.35 [b]	12.51±0.27 [c]	15.06±0.44 [d]
Gallic acid	WS	N.D.	1.92±0.17 [a]	3.15±0.58 [b]	1.27±0.10 [a]	2.81±0.25 [b]
	WD	N.D.	2.07±0.61 [a]	3.03±0.74 [a]	2.95±0.68 [a]	5.49±0.59 [b]
	NA	N.D.	2.81±0.20 [a]	5.43±0.77 [b]	4.75±1.11 [a,b]	8.61±1.53 [c]
	GMD	N.D.	4.05±0.02 [a]	6.27±0.20 [b]	2.14±0.03 [c]	6.40±0.12 [b]
Protocatechuic acid	WS	N.D.	N.D.	N.D.	0.38±0.16 [a]	0.66±0.22 [b]
	WD	N.D.	N.D.	N.D.	0.68±0.25 [a]	0.72±0.18 [a]
	NA	N.D.	N.D.	N.D.	0.95±0.08 [a]	0.86±0.25 [a]
	GMD	N.D.	N.D.	N.D.	1.07±0.21 [a]	1.00±0.40 [a]
vanillic acid	WS	N.D.	0.45±0.02 [a]	0.73±0.02 [a,b]	0.94±0.24 [b]	1.36±0.22 [c]
	WD	N.D.	0.63±0.07 [a]	0.90±0.17 [a,b]	1.20±0.09 [b,c]	1.49±0.34 [c]
	NA	N.D.	0.86±0.28 [a]	1.38±0.41 [a]	1.06±0.17 [a]	1.49±0.51 [a]
	GMD	N.D.	0.85±0.13 [a]	2.28±0.00 [d]	1.11±0.17 [a]	1.63±0.17 [c]
Syringic acid	WS	N.D.	0.42±0.09 [a]	0.66±0.09 [a]	0.99±0.84 [a]	2.22±0.73 [b]
	WD	N.D.	0.40±0.07 [a]	0.75±0.13 [a]	2.03±0.10 [b]	3.19±0.41 [c]
	NA	N.D.	0.82±0.29 [a]	1.39±0.30 [b]	1.98±0.59 [b,c]	2.80±1.03 [c]
	GMD	N.D.	0.57±0.01 [a]	0.75±0.00 [a]	0.95±0.02 [a]	2.74±0.05 [b]
Ellagic acid	WS	N.D.	1.06±0.32 [a]	1.71±0.17 [a]	2.57±0.58 [b]	2.74±0.05 [b]
	WD	N.D.	0.58±0.02 [a]	1.10±0.18 [b]	2.46±0.15 [c]	3.11±0.18 [d]
	NA	N.D.	1.27±0.13 [a]	2.16±0.12 [b]	2.91±0.24 [c]	3.94±0.54 [d]
	GMD	N.D.	0.75±0.00 [a]	1.17±0.01 [b]	0.99±0.06 [b]	1.87±0.01 [c]
∑Hydroxybenzoic acids	WS	N.D.	3.84±0.38 [a]	6.25±0.39 [b]	6.16±0.35 [b]	9.79±0.84 [c]
	WD	N.D.	4.61±0.21 [a]	6.21±0.69 [b]	7.93±0.22 [c]	10.98±1.05 [d]
	NA	N.D.	5.63±1.49 [a]	8.34±1.57 [a,b]	8.34±0.70 [a,b]	10.97±2.28 [b]
	GMD	N.D.	6.22±0.14 [a]	10.47±0.19 [b]	6.26±0.48 [a]	13.64±0.49 [b]

Time (years)		0	Brandy Cask (BC) 1	Brandy Cask (BC) 2	Sherry Cask (SC) 1	Sherry Cask (SC) 2
p-Hydroxy-benzaldehyde	WS	N.D.	N.D.	N.D.	N.D.	N.D.
	WD	N.D.	0.17±0.05 [a]	0.39±0.07 [b]	0.48±0.08 [b]	0.47±0.07 [b]
	NA	N.D.	<LOQ	0.46±0.10 [a]	0.75±0.18 [b]	0.79±0.11 [b]
	GMD	N.D.	N.D.	N.D.	N.D.	N.D.
Syringaldehyde	WS	N.D.	1.35±0.16 [a]	2.01±0.22 [a]	4.03±1.34 [b]	4.80±0.28 [b]
	WD	N.D.	1.48±0.25 [a]	2.39±0.52 [a]	6.52±0.43 [b]	9.89±0.89 [c]
	NA	N.D.	1.72±1.08 [a]	2.56±1.01 [a]	6.52±1.95 [b]	9.03±2.87 [b]
	GMD	N.D.	1.87±0.12 [a]	2.64±0.03 [a]	3.03±0.07 [a]	8.37±0.00 [b]
Vanillin	WS	N.D.	2.22±0.41 [a]	3.45±0.40 [b]	1.66±0.39 [a]	3.35±0.25 [b]
	WD	N.D.	0.84±0.12 [a]	1.54±0.03 [b]	1.97±0.14 [c]	3.00±0.20 [d]
	NA	N.D.	0.90±0.43 [a]	1.51±0.76 [b]	2.29±0.62 [b,c]	3.45±0.99 [c]
	GMD	N.D.	3.07±0.04 [a]	3.90±0.00 [b]	3.24±0.14 [a]	6.87±0.05 [c]
∑Hydroxy-benzaldehydes	WS	N.D.	3.57±0.57 [a]	5.46±0.44 [a,b]	5.69±1.72 [b]	8.15±0.52 [c]
	WD	N.D.	2.32±0.24 [a]	3.93±0.55 [b]	8.49±0.48 [c]	12.89±1.04 [d]
	NA	N.D.	2.63±1.51 [a]	4.07±1.21 [a]	8.81±2.06 [b]	12.47±3.02 [b]
	GMD	N.D.	4.95±0.17 [a]	6.53±0.03 [a]	6.27±0.06 [a]	15.24±0.05 [b]
Coniferylaldehyde	WS	N.D.	0.73±0.16 [a]	0.94±0.14 [a,b]	1.23±0.65 [a,b]	2.29±1.32 [b]
	WD	N.D.	0.64±0.08 [a]	0.81±0.14 [a]	1.39±0.10 [b]	1.88±0.25 [c]
	NA	N.D.	0.70±0.35 [a]	1.03±0.54 [a,b]	1.50±0.47 [a,b]	1.86±0.60 [b]
	GMD	N.D.	0.90±0.02 [a]	1.14±0.02 [a]	1.08±0.02 [a]	2.25±0.00 [b]
Sinapaldehyde	WS	N.D.	1.10±0.28 [a]	1.51±0.31 [a]	3.40±1.53 [a,b]	6.41±3.80 [b]
	WD	N.D.	0.95±0.04 [a]	1.17±0.04 [a]	3.49±0.11 [b]	4.82±0.49 [c]
	NA	N.D.	1.26±0.69 [a]	1.91±1.08 [a,b]	3.66±1.12 [b,c]	4.78±1.51 [c]
	GMD	N.D.	1.67±0.01 [a]	2.20±0.01 [a]	2.47±0.03 [a]	5.36±0.00 [b]
∑Hydroxy-cinnamaldehydes	WS	N.D.	1.83±0.44 [a]	2.45±0.46 [a]	4.63±2.18 [a,b]	8.70±5.11 [b]
	WD	N.D.	1.59±0.10 [a]	1.98±0.17 [a]	4.89±0.10 [b]	6.70±0.71 [c]
	NA	N.D.	1.96±1.03 [a,b]	2.94±0.55 [b,c]	5.16±1.59 [c,d]	6.64±2.11 [d]
	GMD	N.D.	2.57±0.01 [a]	3.34±0.01 [a]	3.56±0.01 [a]	7.61±0.00 [b]
Caffeic acid	WS	N.D.	N.D.	N.D.	0.55±0.03 [a]	0.87±0.28 [b]
	WD	N.D.	N.D.	N.D.	0.41±0.08 [a]	0.41±0.02 [a]
	NA	N.D.	N.D.	N.D.	0.56±0.06 [a]	0.60±0.03 [a]
	GMD	N.D.	N.D.	N.D.	0.33±0.05 [a]	0.55±0.00 [b]
p-coumaric acid	WS	N.D.	N.D.	N.D.	0.55±0.02 [a]	1.12±0.26 [b]
	WD	N.D.	N.D.	N.D.	0.46±0.05 [a]	0.41±0.05 [a]
	NA	N.D.	N.D.	N.D.	0.57±0.07 [a]	0.71±0.11 [b]
	GMD	N.D.	N.D.	N.D.	0.26±0.03 [a]	0.65±0.00 [b]
∑Hydroxycinnamic acids	WS	N.D.	N.D.	N.D.	1.10±0.03 [a]	1.99±0.31 [b]
	WD	N.D.	N.D.	N.D.	0.86±0.07 [a]	0.81±0.06 [a]
	NA	N.D.	N.D.	N.D.	1.13±0.13 [a]	1.31±0.13 [b]
	GMD	N.D.	N.D.	N.D.	0.59±0.03 [a]	1.21±0.00 [b]
**TOTAL** Phenolic compounds and furfural aldehydes	WS	1.92±0.07 [a]	22.72±0.81 [b]	29.01±0.82 [b,c]	21.61±4.66 [b]	34.55±7.47 [c]
	WD	N.D.	9.26±0.44 [a]	13.17±1.51 [b]	24.34±0.78 [c]	33.98±2.37 [d]
	NA	N.D.	11.22±3.85 [a]	16.92±3.06 [a,b]	25.58±3.46 [b,c]	33.89±6.71 [c]
	GMD	6.07±0.00 [a]	37.18±0.26 [b]	45.60±0.56 [c]	29.19±0.12 [d]	52.75±0.98 [e]

Mean ± standard deviation (n=4); A different letter indicates significant differences in a particular row between the values obtained from the Tukey HSD test (p< 0.05). 5-HMF, 5-hydroxymethylfurfural. N.D., non-detected. LOQ, Limit of Quantification.

Table S6. Scores on the **olfactory** sensory descriptors of the samples and analysis of variance of the data for each type of spirit.

SAMPLE	Aromatic intensity	Fruity	Aldehyde	Pungent	Vinous	Herbaceous	Aniseed	Spicy	Vanilla	Oak	Burnt, empyreumatic	Complexity
NA-Initial	2.3±0.5 ^{a}	1.0±0.0	1.5±0.6	2.0±1.2	1.0±0.0 ^{a}	1.0±0.0	1.0±0.0	1.0±0.0 ^{a}	1.0±0.0 ^{a}	1.0±0.0 ^{a}	1.0±0.0	1.3±0.5 ^{a}
NA-BC-1	3.0±0.8 ^{a,b}	1.0±0.0	1.5±0.6	2.8±1.5	1.3±0.5 ^{a}	1.0±0.0	1.0±0.0	2.0±0.0 ^{b}	2.5±0.6 ^{b}	2.3±0.5 ^{b}	1.3±0.5	1.8±0.5 ^{a,b}
NA-BC-2	3.5±0.6 ^{b}	1.0±0.0	1.8±1.0	3.0±1.6	1.3±0.5 ^{a}	1.0±0.0	1.0±0.0	2.3±0.5 ^{b}	2.3±0.5 ^{b}	2.5±0.6 ^{b}	1.5±0.6	2.5±0.6 ^{b,c}
NA-SC-1	3.0±0.0 ^{a,b}	1.3±0.5	1.3±0.5	2.3±0.5	2.5±0.6 ^{b}	1.0±0.0	1.0±0.0	2.3±0.5 ^{b}	2.0±0.0 ^{b}	2.3±0.5 ^{b}	1.3±0.5	2.0±0.0 ^{a,b}
NA-SC-2	3.5±0.6 ^{b}	1.0±0.0	1.8±1.0	2.5±0.6	3.0±0.0 ^{b}	1.0±0.0	1.0±0.0	2.5±0.6 ^{b}	2.5±0.6 ^{b}	2.5±0.6 ^{b}	1.5±0.6	3.0±0.8 ^{c}
p_{NA}	***0.039***	0.438	0.861	0.766	***0.000***			***0.001***	***0.001***	***0.003***	0.573	***0.004***
WD-Initial	3.3±0.5	2.3±1.0	3.0±1.2	3.3±0.5	1.5±0.6 ^{a}	1.0±0.0	1.5±0.6	1.8±0.5 ^{a}	1.0±0.0 ^{a}	1.0±0.0 ^{a}	1.0±0.0	2.0±0.8
WD-BC-1	3.3±1.0	1.5±0.6	3.5±0.6	3.8±0.5	1.3±0.5 ^{a}	1.0±0.0	1.3±0.5	2.3±0.5 ^{a,b}	2.3±0.5 ^{b}	2.5±0.6 ^{b}	1.5±0.6	2.5±0.6
WD-BC-2	3.8±1.0	1.5±0.6	3.0±0.8	4.0±0.8	1.8±1.0 ^{a}	1.0±0.0	1.5±0.6	2.8±0.5 ^{b}	2.5±0.6 ^{b}	3.0±0.0 ^{b}	1.5±0.6	3.0±0.8
WD-SC-1	3.8±0.5	1.5±0.6	3.5±0.6	3.5±0.6	2.8±0.5 ^{b}	1.0±0.0	1.0±0.0	2.8±0.5 ^{b}	2.5±0.6 ^{b}	2.5±0.6 ^{b}	1.5±0.6	2.8±0.5
WD-SC-2	3.8±1.3	1.3±0.5	3.3±1.5	3.5±0.6	3.3±0.5 ^{b}	1.0±0.0	1.5±0.6	2.5±0.6 ^{a,b}	3.0±0.8 ^{b}	2.8±1.0 ^{b}	1.5±0.6	3.0±0.0
p_{WD}	0.817	0.305	0.903	0.496	***0.002***		0.544	***0.056***	***0.002***	***0.001***	0.573	0.176
WS-Initial	3.5±1.0	2.8±0.5	2.8±0.5	2.5±0.6	1.3±0.5 ^{a}	1.0±0.0	2.5±1.0	2.0±0.8	1.0±0.0 ^{a}	1.0±0.0 ^{a}	1.0±0.0	2.0±0.8 ^{a}
WS-BC-1	4.0±0.0	2.0±0.0	2.3±0.5	3.3±0.5	1.0±0.0 ^{a}	1.0±0.0	2.0±0.0	3.0±0.0	2.8±0.5 ^{b}	3.0±0.0 ^{b}	1.5±0.6	2.8±0.5 ^{a,b}
WS-BC-2	3.8±1.3	2.3±0.5	2.5±0.6	2.8±0.5	1.3±0.5 ^{a}	1.0±0.0	2.0±0.0	2.5±0.6	2.8±0.5 ^{b}	3.5±0.6 ^{b}	1.5±0.6	3.0±0.8 ^{b}
WS-SC-1	3.8±0.5	2.3±0.5	2.0±0.0	2.8±0.5	3.3±0.5 ^{b}	1.0±0.0	2.3±0.5	2.8±0.5	2.8±1.0 ^{b}	3.0±0.8 ^{b}	1.5±0.6	3.3±0.5 ^{b}
WS-SC-2	3.8±1.3	2.3±0.5	2.5±0.6	2.5±1.0	3.5±1.3 ^{b}	1.0±0.0	2.0±0.8	3.3±1.0	3.3±1.7 ^{b}	3.3±1.0 ^{b}	1.5±0.6	3.5±0.6 ^{b}
p_{WS}	0.964	0.252	0.284	0.488	***0.000***		0.721	0.127	***0.034***	***0.000***	0.573	***0.050***
GMS-Initial	4.0±0.8	2.8±0.5	4.3±1.0	4.0±0.8	1.5±0.6 ^{a}	4.3±1.0	2.8±1.5	2.3±0.5	1.0±0.0 ^{a}	1.0±0.0 ^{a}	1.8±0.5	2.0±1.2
GMS-BC-1	3.8±0.5	2.0±0.0	3.5±0.6	3.5±0.6	1.5±0.6 ^{a}	3.5±0.6	3.0±1.2	2.8±0.5	2.0±0.0 ^{a,b}	2.3±0.5 ^{b}	1.8±0.5	2.5±0.6
GMS-BC-2	4.3±1.0	2.5±0.6	3.8±1.0	3.8±1.5	1.8±1.0 ^{a,b}	3.5±1.0	2.8±1.0	2.8±1.0	2.5±0.6 ^{b}	2.8±1.0 ^{b}	1.8±0.5	3.0±0.8
GMS-SC-1	3.8±0.5	2.0±0.8	3.3±1.0	3.5±1.0	2.8±0.5 ^{b,c}	3.3±1.0	2.5±0.6	3.0±0.8	2.3±0.5 ^{b}	2.5±0.6 ^{b}	1.8±0.5	3.3±0.5
GMS-SC-2	4.5±0.6	2.5±0.6	3.5±1.0	4.3±1.0	3.8±1.0 ^{c}	3.3±1.5	2.5±1.7	3.3±1.5	2.5±1.3 ^{b}	3.0±1.2 ^{b}	1.8±0.5	3.5±1.0
p_{MD}	0.499	0.275	0.601	0.798	***0.002***	0.653	0.976	0.646	***0.035***	***0.017***	1.000	0.143

Mean ± standard deviation; A different letter indicates significant differences in a particular row between the values obtained from the Tukey HSD test ($p < 0.05$). N.D., non-detected. LOQ, limit of quantification.

Table S7. Scores on the **olfactory-gustatory** sensory descriptors of the samples and analysis of variance of the data for each type of spirit.

SAMPLE	Alcohol	Acidity	Bitterness	Sweetness	Astringency	Oak	Complexity	Smoothness	Balance	Persistence
NA-Initial	2.8±1.0	1.3±0.5 ^{a}	1.0±0.0 ^{a}	1.8±0.5 ^{b}	1.0±0.0 ^{a}	1.0±0.0 ^{a}	1.0±0.0 ^{a}	2.8±0.5	1.5±0.6 ^{a}	1.3±0.6 ^{a}
NA-BC-1	2.8±1.0	1.5±0.6 ^{a,b}	1.5±0.6 ^{a,b}	1.0±0.0 ^{a}	2.0±0.0 ^{b}	2.5±0.6 ^{b}	1.8±1 ^{a,b}	2.0±1.2	2.0±0.0 ^{a,b}	2.3±0.5 ^{b}
NA-BC-2	4.0±0.8	2.3±0.5 ^{b}	2.5±0.6 ^{c}	1.0±0.0 ^{a}	2.3±0.5 ^{b}	2.5±0.6 ^{b}	2.5±0.6 ^{b}	2.0±0.8	2.0±0.0 ^{a,b}	2.3±0.5 ^{b}
NA-SC-1	3.3±0.5	1.8±0.5 ^{a,b}	2.3±0.5 ^{b,c}	1.3±0.5 ^{a}	2.3±0.5 ^{b}	2.0±0.0 ^{b}	2.3±0.5 ^{b}	2.0±0.8	2.3±0.5 ^{b,c}	2.0±0.0 ^{a,b}
NA-SC-2	3.0±0.0	2.3±0.5 ^{b}	2.0±0.8 ^{b,c}	1.0±0.0 ^{a}	2.0±0.8 ^{b}	2.5±0.6 ^{b}	2.3±0.5 ^{b}	2.5±0.6	2.8±0.5 ^{c}	2.5±0.6 ^{b}
p_{NA}	0.154	***0.043***	***0.013***	***0.017***	***0.013***	***0.001***	***0.020***	0.562	***0.010***	***0.057***
WD-Initial	3.0±1.2	2.0±0.8	1.3±0.5 ^{a}	1.5±0.6 ^{b}	1.0±0.0 ^{a}	1.0±0.0 ^{a}	1.5±0.6 ^{a}	2.8±1.0	1.8±0.5 ^{a}	1.5±0.6 ^{a}
WD-BC-1	3.0±0.8	2.0±0.8	1.8±1.0 ^{a,b}	1.0±0.0 ^{a}	2.0±0.8 ^{b}	2.5±0.6 ^{b}	1.8±0.5 ^{a}	1.8±0.5	1.8±0.5 ^{a}	2.0±0.0 ^{a,b}
WD-BC-2	3.3±1.0	2.0±0.8	2.3±0.5 ^{b,c}	1.0±0.0 ^{a}	2.5±0.6 ^{b}	2.8±0.5 ^{b}	2.8±0.5 ^{b}	2.0±0.8	2.5±0.6 ^{a,b}	2.0±0.0 ^{a,b}
WD-SC-1	2.8±1.0	2.3±0.5	2.0±0.0 ^{a,b,c}	1.0±0.0 ^{a}	2.0±0.0 ^{b}	2.8±0.5 ^{b}	2.8±0.5 ^{b}	2.0±0.8	2.8±0.5 ^{b}	2.8±0.5 ^{c}
WD-SC-2	3.0±0.0	2.0±0.0	2.8±0.5 ^{c}	1.0±0.0 ^{a}	2.5±0.6 ^{b}	2.8±0.5 ^{b}	2.8±1.0 ^{b}	2.3±1.0	2.8±0.5 ^{b}	2.3±0.5 ^{b,c}
p_{WD}	0.954	0.977	***0.026***	***0.043***	***0.006***	***0.000***	***0.023***	0.520	***0.022***	***0.010***
WS-Initial	4.0±0.0 ^{c}	2.3±1.0	1.5±0.6	1.3±0.5	1.0±0.0 ^{a}	1.0±0.0 ^{a}	2.5±0.6	2.3±0.5 ^{a,b}	1.8±1.0	2.0±0.8
WS-BC-1	3.8±0.5 ^{b,c}	2.0±0.8	2.3±0.5	1.0±0.0	2.3±1.0 ^{b}	3.0±0.0 ^{b,c}	2.8±0.5	1.5±0.6 ^{a}	2.5±0.6	2.3±1.0
WS-BC-2	3.0±0.8 ^{a,b}	2.5±0.6	2.0±0.8	1.3±0.5	2.3±1.0 ^{b}	3.8±0.5 ^{c}	2.5±0.6	2.0±0.0 ^{a}	3.0±0.8	3.0±0.0
WS-SC-1	2.5±0.6 ^{a}	1.5±0.6	2.0±0.8	1.5±0.6	2.0±0.8 ^{a,b}	2.8±0.5 ^{b}	2.5±0.6	3.0±0.0 ^{b}	2.8±1.0	2.8±0.5
WS-SC-2	3.0±0.8 ^{a,b}	2.8±0.5	2.5±0.6	1.0±0.0	2.8±1.0 ^{b}	3.3±1 ^{b,c}	2.8±0.5	2.0±0.8 ^{a}	3.0±0.8	3.0±0.8
p_{WS}	**0.023**	0.172	0.343	0.415	**0.051**	***0.000***	0.905	***0.011***	0.242	0.214
GMS-Initial	4±0	3±0	1.8±1.0	1.3±0.5	1.8±1.0	1.0±0.0 ^{a}	2.0±0.8	1.8±1.0	2.0±1.4	2.5±1.3
GMS-BC-1	3.8±0.5	2.8±0.5	2.5±0.6	1.0±0.0	2.5±0.6	2.5±0.6 ^{b}	2.5±1.0	1.5±0.6	2.0±0.8	3.0±0.0
GMS-BC-2	3.8±1.3	3.0±0.0	2.3±1.3	1.0±0.0	2.5±0.6	3.0±0.8 ^{b}	2.8±0.5	1.8±1.0	2.8±1.0	2.5±1.0
GMS-SC-1	4±0	2.5±0.6	2.8±0.5	1.0±0.0	2.5±0.6	2.5±0.6 ^{b}	2.8±0.5	2.0±0.8	2.8±0.5	3.0±0.0
GMS-SC-2	3.5±0.6	2.5±0.6	2.3±1.0	1.0±0.0	2.8±0.5	3.0±0.8 ^{b}	3.0±0.8	2.0±1.2	3.3±1.3	3.3±1.3
p_{MD}	0.803	0.293	0.612	0.438	0.305	***0.003***	0.431	0.929	0.399	0.716

Mean ± standard deviation; A different letter indicates significant differences in a particular row between the values obtained from the Tukey HSD test ($p < 0.05$). N.D., non-detected. LOQ, limit of quantification.
